# Adopting a cultural lens on social capital of older Chinese and South Asians in Hong Kong: A comparative CFA study

**DOI:** 10.1093/geroni/igaf122.236

**Published:** 2025-12-31

**Authors:** Codiez Z D Huang, Daniel W L Lai, Alison Y T Ou, Vincent W P Lee, Jessica J Li, Shireen Surood, Doris S F Yu, Gary K K Lau

**Affiliations:** Hong Kong Baptist University, Kowloon, Hong Kong, China (People’s Republic); Hong Kong Baptist University, Kowloon, Hong Kong, China (People’s Republic); Hong Kong Baptist University, Kowloon, Hong Kong, China (People’s Republic); Hong Kong Baptist University, Kowloon, Hong Kong, China (People’s Republic); The Chinese University of Hong Kong, Hong Kong, China (People’s Republic); Island Health, Victoria, British Columbia, Canada; The University of Hong Kong, Hong Kong, China (People’s Republic); The University of Hong Kong, Hong Kong, China (People’s Republic)

## Abstract

Social capital structures play an important role in addressing social and health care needs of older adults. Knowing culture provides the context for values, beliefs and behaviors, it is imperative to use an ethnically-specific approach to understand the complexity of social capital structures in a multi-national population. Ethnic diversity is a prominent characteristic among the aging population in Hong Kong, with the South Asian aged 55 increased at almost three times the rate of the local aged cohort. This study aimed to examined how social capital structures determined their care needs among these diverse group. This study conducted telephone survey to 800 Hong Kong older adults via random digital dialing. Another purposive sample of 215 South Asians were interviewed face-to-face. Confirmatory Factor Analysis revealed notable differences in social capital structures between the groups, with both models showing excellent reliability (South Asians: CFI=0.999, TLI=0.999, RMSEA=0.078; Hong Kong: CFI=0.991, TLI=0.987, RMSEA=0.077). South Asian older adults emphasized neighborhood cohesion, community interactions, and collective support, reflecting collectivist cultural values. Conversely, Hong Kong older adults focused on individual community participation, family-oriented support, and trust networks from family to neighbors, indicative of a more individualistic cultural orientation centered on personal achievements and family ties. These findings highlight the need for culturally responsive social policies. Programs for South Asian older adults should enhance neighborhood ties and collective support, while initiatives for Hong Kong older adults should strengthen familial networks and personalized social engagement. Future research should explore these distinctions to guide inclusive aging policies and community interventions.

